# Dynamic Imaging of Individual Remyelination Profiles in Multiple Sclerosis

**DOI:** 10.1002/ana.24620

**Published:** 2016-05-06

**Authors:** Benedetta Bodini, Mattia Veronese, Daniel García‐Lorenzo, Marco Battaglini, Emilie Poirion, Audrey Chardain, Léorah Freeman, Céline Louapre, Maya Tchikviladze, Caroline Papeix, Frédéric Dollé, Bernard Zalc, Catherine Lubetzki, Michel Bottlaender, Federico Turkheimer, Bruno Stankoff

**Affiliations:** ^1^ Sorbonne UniversitésUPMC University Paris 06UMR S 1127, and CNRS UMR 7225, and ICMF‐75013ParisFrance; ^2^ Department of Neuroimaging, Institute of PsychiatryKing's College LondonLondonUnited Kingdom; ^3^ Service Hospitalier Fréderic Joliot, SHFJ, I2BM‐DSVCEAOrsayFrance; ^4^ Department of Behavioral and Neurological SciencesUniversity of SienaSienaItaly; ^5^ APHP Hôpital Saint‐AntoineParisFrance; ^6^ APHPHopital Pitié-SalpetrièreParisFrance

## Abstract

**Background:**

Quantitative in vivo imaging of myelin loss and repair in patients with multiple sclerosis (MS) is essential to understand the pathogenesis of the disease and to evaluate promyelinating therapies. Selectively binding myelin in the central nervous system white matter, Pittsburgh compound B ([^11^C]PiB) can be used as a positron emission tomography (PET) tracer to explore myelin dynamics in MS.

**Methods:**

Patients with active relapsing‐remitting MS (n = 20) and healthy controls (n = 8) were included in a longitudinal trial combining PET with [^11^C]PiB and magnetic resonance imaging. Voxel‐wise maps of [^11^C]PiB distribution volume ratio, reflecting myelin content, were derived. Three dynamic indices were calculated for each patient: the global index of myelin content change; the index of demyelination; and the index of remyelination.

**Results:**

At baseline, there was a progressive reduction in [^11^C]PiB binding from the normal‐appearing white matter to MS lesions, reflecting a decline in myelin content. White matter lesions were characterized by a centripetal decrease in the tracer binding at the voxel level. During follow‐up, high between‐patient variability was found for all indices of myelin content change. Dynamic remyelination was inversely correlated with clinical disability (*p* = 0.006 and beta‐coefficient = –0.67 with the Expanded Disability Status Scale; *p* = 0.003 and beta‐coefficient = –0.68 with the MS Severity Scale), whereas no significant clinical correlation was found for the demyelination index.

**Interpretation:**

[^11^C]PiB PET allows quantification of myelin dynamics in MS and enables stratification of patients depending on their individual remyelination potential, which significantly correlates with clinical disability. This technique should be considered to assess novel promyelinating drugs. Ann Neurol 2016;79:726–738

As the leading cause of onset of neurological disability in young adulthood, multiple sclerosis (MS) presents an enormous social and economic burden in the Western world.^1^ MS pathophysiology predominantly involves autoimmune aggression of central nervous system (CNS) myelin sheaths, resulting in inflammatory demyelinating lesions and subsequent irreversible axonal degeneration. Considerable efforts have been made over past decades to develop immunoactive therapies. These have shown significant effects in reducing the number of clinical relapses; however, they have failed to demonstrate any efficacy in reducing or delaying long‐term disability progression.^2^ We are therefore assisting to a shift in therapeutic objectives from the development of new immune drugs toward the identification of therapeutic strategies to promote myelin regeneration, an endogenous process that is expected to restore secure and rapid conduction as well as to protect axons from degeneration.^3^

In animal models, myelin regeneration is a very effective process that is activated by default in response to any sort of myelin damage, resulting in efficient reconstruction of the area of myelin loss.^4^ To date, little is known about the dynamics of remyelination in patients with MS over the course of their disease. Sensitive and specific imaging tools designed to measure myelin in vivo are essential to understand how and why spontaneous remyelination succeeds or fails in MS, as well as to quantify the potential effects of new promyelinating therapies.

Advanced magnetic resonance imaging (MRI) sequences, such as magnetization transfer imaging, diffusion‐weighted imaging, and T2 relaxometry, which are able to generate quantitative images exploiting physical properties of the brain parenchyma, have been proposed to gain indirect information about the myelin compartment in the human brain.^5^ However, these techniques are not specific for myelin because they are affected to various extents by intra‐ and extracellular water, axons, edema, and inflammatory infiltration. Positron emission tomography (PET), which allows selective targets to be marked with radiolabeled compounds, is a promising alternative for myelin imaging. Following the pilot demonstration indicating that the stilbene Congo red derivative 1,4‐bis(p‐aminostyryl)‐2‐methoxy benzene, could be used as a myelin tracer suitable for PET imaging,^6^ a similar affinity for myelin was reported for other stilbene derivatives.^7^, ^8^, ^9^, ^10^ These tracers, all previously known as amyloid markers, were hypothesized to bind to proteins characterized by a similar conformation contained in amyloid plaques and myelin.^11^, ^12^ On this basis, Pittsburgh compound B (PiB), a thioflavin compound binding to amyloid plaques, was also identified as a promising myelin tracer suitable for human PET studies.^13^ In rodent demyelinating lesions, microPET with [^11^C]PiB showed great sensitivity in capturing remyelination after demyelination.^10^ Preliminary data obtained from humans further demonstrated that [^11^C]PiB PET was sensitive enough to detect myelin loss in MS lesions.^13^ A noninvasive parametric voxel‐wise quantification procedure based on the extraction of reference regions using a supervised clustering algorithm showing higher reproducibility compared to previously used semiquantitative methods, has recently been shown to allow reliable longitudinal evaluation of [^11^C]PiB binding in the white matter (WM) of healthy volunteers.^14^

Here, we report the results of the first longitudinal study in which PET with [^11^C]PiB was used to quantify in vivo myelin loss and regeneration in the WM lesions of patients with MS and to explore the clinical relevance of these processes.

## Subjects and Methods

## Subjects

Twenty patients with relapsing‐remitting MS according to the revised McDonald criteria^15^ with at least one gadolinium‐enhancing (Gd+) lesion (defined as all voxels localized inside a ring‐enhancing lesion) with an in‐plane maximum diameter larger than 6 mm on MRI at study entry (13 women; mean age: 32.3 years; standard deviation [SD]: 5.6) and an age‐ and sex‐matched group of 8 healthy volunteers (5 women; mean age: 31.6 years; SD, 6.3) signed written informed consent to participate in a clinical imaging protocol approved by the local ethics committee (Table 1).

**Table 1:** Demographic, Clinical, and Radiological Characteristics of Patients and Healthy Controls at Study Entry

Clinical and Radiological Characteristics	Patients	Healthy Volunteers
Number	20	8
Age, mean ± SD	32.31 ± 5.71	31.57 ± 6.37
Gender, female/male	13/7	5/3
Disease duration, mean ± SD	7.45 ± 5.77	—
EDSS, median (range)	2 (0–6)	—
MSSS, median (range)	3.43 (0.45–6.92)	—
Treatment at study entry, no. of patients	No treatment = 4 First‐line treatment = 10 Second‐line treatment = 6	—
T2 lesion load, cc, mean ± SD	109.79 ± 73.05	—
Black hole lesion load, cc, mean ± SD	9.3 ± 11.26	—
Gadolinium‐enhancing lesions, cc, mean ± SD	4.46 ± 4.02	—

EDSS = Expanded Disability Status Scale; MSSS = Multiple Sclerosis Severity Scale; SD = standard deviation.

### Study Design

At inclusion, all patients were clinically assessed and scored using the Expanded Disability Status Scale^16^ (EDSS) and the Multiple Sclerosis Severity Scale^17^ (MSSS), which is designed to provide a measure of disease severity by adding the element of disease duration to the EDSS; all patients underwent MRI and PET scan. The 19 patients who completed the study (1 patient withdrew from the study after the first PET scan because of personal reasons) were randomly assigned to two subgroups to repeat the whole protocol after either 1 to 2 months (n = 9) or 3 to 4 months (n = 10) after inclusion to explore the best time interval in which to capture and quantify dynamic remyelination and demyelination. All healthy volunteers underwent a second PET scan 1 month after inclusion. No adverse event was observed during the study.

### Image Acquisition and Analysis

#### PET Images

**Figure 1:**
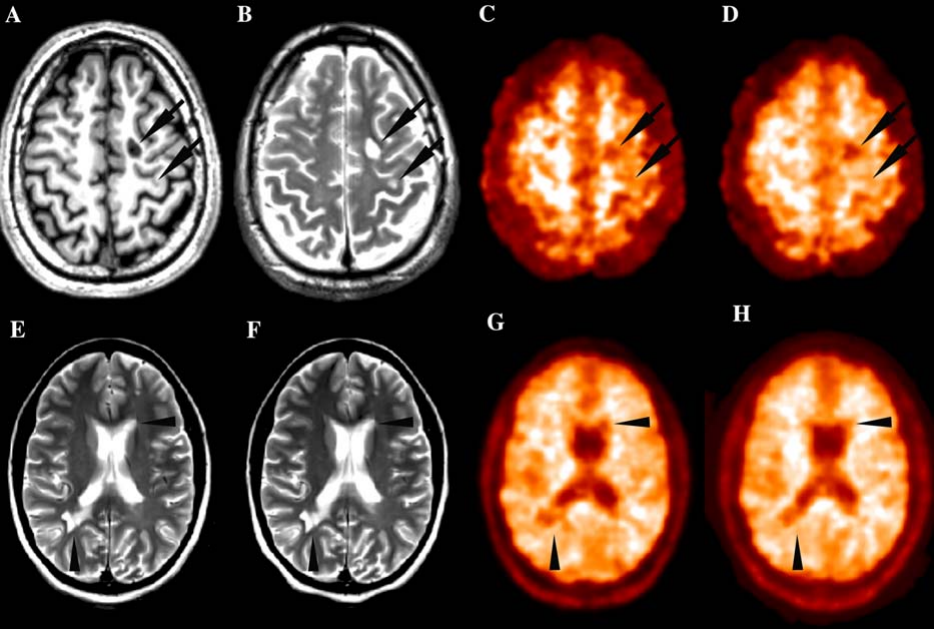
Representative magnetic resonance imaging (MRI) and positron emission tomography (PET) images from MS patients. (A–D) T1‐weighted MRI (A), T2‐weighted MRI (B), Pittsburgh compound B ([^11^C]PiB) standard uptake value (SUV) map (C), and [^11^C]PiB distribution volume ratio (DVR) parametric map (D) of a single patient at study entry. SUV maps are semi‐quantitative measures of the tracer's uptake obtained by averaging the PET frames acquired between the minutes 30 and 70 of the examination and correcting the values for the tracer's injected dose and the patient's weight. DVR maps are quantitative parametric maps obtained with the automatic extraction of a reference region and the subsequent application of the Logan graphical method. Arrows indicate two typical multiple sclerosis white matter lesions appearing as areas of decreased uptake both on SUV and DVR images. T2‐weighed MRI at study entry (E) and after 3 months (F) and [^11^C]PiB DVR parametric map at baseline (G) and at follow‐up (H) of a single patient. Arrowheads (G and H) indicate two lesions visible on MRI scans that appear as regions of decreased DVR values on PET images and point to parts of the lesions where a subtle local increase in DVR value between the first and second PET scan is visible, suggesting local myelin regeneration developing during follow‐up. Note that the same lesion appears unchanged on T2‐weighed images.

Image acquisition, reconstruction, and quantification were performed as recently described.^14^ Briefly, PET examinations were performed on a high‐resolution research tomograph (HRRT; CPS Innovations, Knoxville, TN), which achieves an intraslice spatial resolution of ∼2.5mm full width at half maximum, with 25‐cm axial and 31.2‐cm transaxial fields of view. The 90‐minute emission scan was initiated with a 1‐minute intravenous bolus injection of [^11^C]PiB (mean = 358 ± 34 MBq). Images were reconstructed using the three‐dimensional (3D) ordinary Poisson ordered subset expectation maximization algorithm with 10 iterations (considered to represent the appropriate trade‐off between image resolution, quality of the data, and reliability of results).^14^ An additional smoothing filter implementing the point spread function, which has been shown to be effective in reducing the effect of partial volume in PET data,^18^ was applied to the 10‐iteration reconstructed image. All the resulting dynamic PET images consisted of 25 time interval (time frames) images: six 1‐minute frames for the initial 6 minutes (6×1), followed by 6×2‐, 4×3‐, 6×5‐, and 3×10‐minute frames, with a voxel size of 1.22 × 1.22 × 1.22 mm. Interframe subject motion correction was applied by realigning each PET frame to a common reference space by using a previously described procedure.^19^ Data were also corrected for carbon‐11 decay. To avoid blood sampling in our patient cohort, we chose to quantify our PET scans using a reference region approach. A supervised clustering method already validated for [^11^C]PiB was used for the extraction of the reference region time activity curve (TAC).^14^, ^20^, ^21^ Briefly, this method consists of the multiple regression of all PET image voxel TACs on a predefined set of kinetic classes. After regression, all the voxels associated with the reference class with probability >90% are combined and the average of their TACs defines the reference input function. As described in the validation study performed using healthy controls, three classes were defined: normal gray matter (GM); high specific binding GM; and blood pool. The reference class of choice was normal GM.^14^, ^21^ Once the reference TAC was extracted, [^11^C]PiB binding was calculated by applying a kinetic operator to the measured dynamic PET activity during the last 60 minutes of the PET acquisition. The Logan graphical reference method^22^ was then applied at the voxel level on PET scans in native space and returned parametric maps of [^11^C]PiB binding measured as the distribution volume ratio (DVR; defined as the ratio of the total distribution volume between the target and the reference region; Fig 1).

#### MR Images

MR images were collected using a 3 Tesla Siemens TRIO 32‐channel TIM system, and included a 3D T1‐weighted magnetization‐prepared rapid gradient‐echo (MPRAGE), two‐dimensional proton density, T2‐weighted (T2‐w) imaging, 3D fluid‐attenuated inversion recovery (FLAIR), and pre‐ and postgadolinium T1 spin‐echo sequences (Supplementary Table 1).

##### Native Space Postprocessing

For each patient, T2‐w and T1 spin‐echo images were registered onto the corresponding 3D‐T1 MPRAGE image using a fully affine transformation (12 parameters; performed with FLIRT, FMRIB's Linear Image Registration Tool, which is part of FSL, FMRIB's Software Library; http://fsl.fmrib.ox.ac.uk/fsl/fslwiki/), and the derived transforms were used to align lesion masks with the individual 3D‐T1 MPRAGE scans. After performing a “lesion‐filling” procedure,^23^ 3D‐T1 MPRAGE voxels were classified as WM, GM, and cerebrospinal fluid (CSF) using the SPM8 software (Statistical Parametric Mapping Version 8; http://www.fil.ion.ucl.ac.uk/spm/software/spm8/) when the probability of belonging to each tissue was more than 90%. On each subject's 3D‐T1 MPRAGE scan, the following regions of interest (ROIs) were defined by a single experienced observer (B.B.): (1) GM; (2) normal‐appearing WM (NAWM), defined as the WM outside visible lesions; (3) perilesional WM, defined as the 2‐mm in‐plane voxel rim of NAWM surrounding T2‐w lesions; (4) WM lesions, identified on T2‐w scans; (5) black holes, defined on T1‐weighted spin‐echo scans as hypointense areas compared to the NAWM; and (6) Gd+ lesions. In healthy controls, WM and GM were defined. To further minimize the impact of the partial volume effect, which is known to be inversely proportional to the dimension of any given ROI, only lesions with a minimal diameter of 2.5mm (corresponding to the approximate resolution of the HRRT camera) were retained for further analysis.

##### Standard Space Postprocessing

For each patient, the 3D‐T1 MPRAGE scans acquired at baseline and at follow‐up were first registered one onto the other with linear transformation, using FLIRT. The derived transforms were then deconstructed into two half‐way transforms, which placed the two original images onto a subject‐specific half‐way space so that they both suffered the same amount of interpolation‐related blurring. The average image of the two half‐way 3D‐T1 MPRAGE scans was then registered onto a standard brain image (MNI152) using a non‐linear transformation (performed with FNIRT, FMRIB's Non‐Linear Image Registration Tool, which is also part of FSL, FMRIB's Software Library; http://fsl.fmrib.ox.ac.uk/fsl/fslwiki/). By linking derived transforms, all the previously generated ROIs were taken from the original 3D‐T1 MPRAGE scans in native space and placed in standard space.

In controls, 3D‐T1 MPRAGE scans in native space were directly registered on a standard brain image (MNI152) using a nonlinear transformation performed with FNIRT, and the derived transforms were used to place ROIs in standard space.

#### PET‐MRI Coregistration

For all subjects, each DVR parametric map was linearly registered onto the corresponding 3D‐T1 MPRAGE scan with FLIRT; the derived transform was first inverted and then used to align all ROIs with the DVR parametric map in native space at both time points.

Then, the previously generated transforms were linked with one another to align each DVR parametric map in native space to the standard brain image. They were then used to take all ROIs to the DVR parametric maps in standard space.

#### Indices of Myelin Content Change

The mean and SD of [^11^C]PiB binding were computed for each subject and for each ROI from DVR parametric maps in native space at baseline.

Demyelinated voxels inside patients' T2‐w lesion masks at baseline were defined on PET scans at study entry once registered to standard space, based on local thresholds in comparison with the PET scans of healthy controls after correction for the effect that the distance from the CSF has on local DVR values to minimize partial volume effects.^14^ In particular, one given lesional voxel in patients was defined as demyelinated if its DVR value was ≤1 SD below the mean DVR value of all the voxels in healthy controls that were localized at the same distance from the CSF as the given voxel (and were therefore potentially affected to the same extent by partial volume). The percentage of demyelinated voxels over the total T2‐w lesion load measured at baseline was calculated from each patient's individual DVR map at both time points in standard space.

The difference between the derived percentage at the second time point and the corresponding percentage at baseline was defined as the *global index of myelin content change* and reflected the subject‐specific prevalence of either myelin loss (positive values) or myelin repair (negative values) over the follow‐up interval. The *index of dynamic demyelination*, reflecting ongoing myelin loss, was defined as the proportion of normally myelinated voxels at baseline that were classified as demyelinated at the second time point. The *index of dynamic remyelination*, which reflected ongoing myelin repair, was defined as the proportion of lesional voxels classified as demyelinated at baseline and reached a myelin level within normal limits at the second time point.

### Statistical Analysis

#### Baseline group‐level analysis

To test for differences in mean DVR at baseline between patients' NAWM and GM and healthy volunteers' WM and GM, respectively, two multiple linear regressions were used, with group as the factor of interest and gender and age as covariates. A mixed‐effect linear model was used to test for differences in mean DVR between NAWM and PWM, T2‐w lesions, Gd+ lesions, and black holes, which included the subject as the random effect and age, gender, disease duration, and T2‐w lesion load as covariates. The reduction in mean DVR between two ROIs was calculated and reported as the percent reduction in mean DVR.

To investigate the effect of each lesional voxel's distance in millimeters to the nearest point on the lesional border on its corresponding DVR value, a mixed‐effect linear model was used in which the subject was included as the random effect, and age and gender were included as covariates.

#### Clinical Correlations

The correlation between the PET‐derived indices of myelin content change and clinical scores was calculated using two separate multiple linear regressions: the first with absolute values of EDSS and the second with absolute values of MSSS as response variables, and age, gender, and T2‐w lesion load as additional covariates. Given that three regressions were run for each of the two clinical variables (one for each of the three indices of myelin content change), a Bonferroni‐adjusted significance level for these tests has been set at *p* = 0.017. To confirm significant results, ordinal regressions were also performed using a proportional‐odds cumulative logit model, where EDSS and MSSS values were classified in three groups according to disease severity (mild disability = EDSS ≤1.5 or MSSS ≤3; moderate disability = EDSS 2–3 or MSSS 3–6; severe disability = EDSS >3 or MSSS >6) and entered as dependent variables, and age, gender, and T2‐w lesion load were included as covariates. An exploratory post‐hoc analysis was also performed using significant models, repeating regressions after including treatment status at study entry as an additional covariate.

#### Identification of Potential Contributors to the Indices of Dynamic Demyelination and Remyelination

Age, gender, disease duration, temporal distance between the two PET scans, treatment at study entry, and volume of T2‐w and Gd+ lesion load were considered potential contributors and were included as independent variables in two separate multiple linear regressions, in which the indices of dynamic demyelination and remyelination were entered as dependent variables.

Unless specified otherwise, results are reported as significant at *p* < 0.05. Regression coefficients have been calculated as the mean change in the dependent variable for one unit of change in the predictor variable while holding other predictors in the model constant, and are reported below as “coefficients.”

## Results

### Nonlesional WM and GM [^11^C]PiB Binding Does Not Differ Between MS Patients and Healthy Controls

In patients, mean DVR for NAWM was 1.25 (SD = 0.05), whereas mean DVR for GM was 1.09 (SD = 0.01). In healthy volunteers, mean DVR was 1.25 (SD = 0.04) for WM and 1.09 (SD = 0.01) for GM, respectively. No significant difference in mean DVR was found between NAWM in patients and WM in healthy controls (coefficient = 0.003; *p* = 0.893; 95% confidence interval [CI] = –0.044 to 0.050) or between GM in patients and GM in healthy volunteers (coefficient = –0.008; *p* = 0.081; 95% CI = –0.017 to 0.001).

### Demyelination at Baseline: A Gradient of [^11^C]PiB Binding Decrease From Normal‐Appearing Tissue to the Center of the Lesion

**Figure 2:**
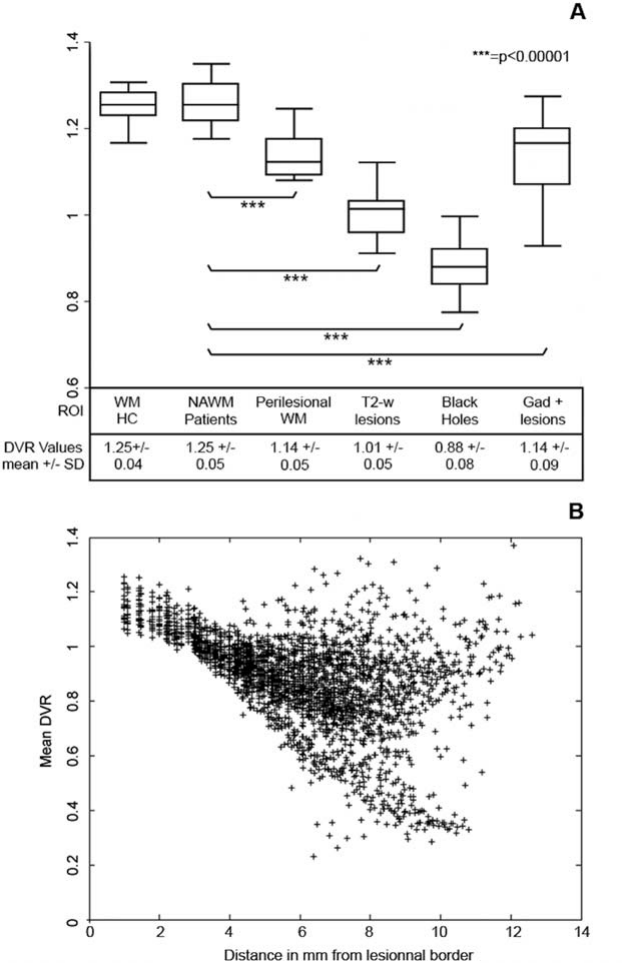
Gradient in Pittsburgh compound B ([^11^C]PiB) binding from normal‐appearing WM to the center of lesions. (A) Box plot diagrams showing the median DVR (middle line) and range for each ROI at baseline in healthy controls and patients (from left to right: WM in healthy controls, normal‐appearing WM in patients, perilesional WM, T2‐weighted lesions, black holes, and gadolinium‐enhancing lesions). These box plots show that the lowest myelin content was detected in the “black holes”, the hypointense lesions on T1 spin‐echo scans that are known to represent the most severely demyelinated lesions in MS brains. A paired *t* test was used in a within‐patient analysis to test for between‐region differences in myelin content. (B) [^11^C]PiB binding values are negatively correlated with the distance from the lesional border. Each point in this scatter plot diagram represents the mean DVR value (y axis) of all the voxels localized at any given distance in millimeters from the lesional border (x axis) in any given patient. Although voxels closer to the lesional border, on average, present higher myelin content values, those located far from the lesional border tend to present lower myelin content values. The correlation between each voxel's distance in millimeters from the lesional border and its corresponding DVR value, which was tested using a mixed‐effect linear model in which the subject was included as random effect and age and gender were covariates, was highly significant (*p* = 0.00001). DVR = distribution volume ratio; MS = multiple sclerosis; NAWM = normal‐appearing white matter; ROI = region of interest; WM = white matter. SD = standard deviation; T2‐w = T2‐weighted.

Compared to NAWM in patients, mean DVR values were significantly lower in perilesional WM (percent reduction in mean DVR = –9.84%; *p* < 0.0001), T2 lesions (percent reduction in mean DVR = –20.24%; *p* < 0.0001), Gd+ lesions (percent reduction in mean DVR = –9.52%; *p* < 0.0001), and black holes (percent reduction in mean DVR = –30.95%; *p* < 0.0001; Fig 2A; Supplementary Table 2).

In T2‐w, black holes, and Gd+ lesions, the mean percentage of demyelinated voxels over total lesion load were 53.5% (SD = 9.1), 68.1% (SD = 11.7), and 25.9% (SD = 15.4), respectively.

A negative correlation between the distance of voxels from the corresponding lesional border and their DVR value was found (coefficient = –0.002; 95% CI = –0.025 to –0.016; *p* < 0.0001), with lower DVR values for the center of the lesions and higher DVR values for the periphery (Fig 2B).

### Between‐Patient Variability in Myelin Loss and Regeneration Levels

**Figure 3:**
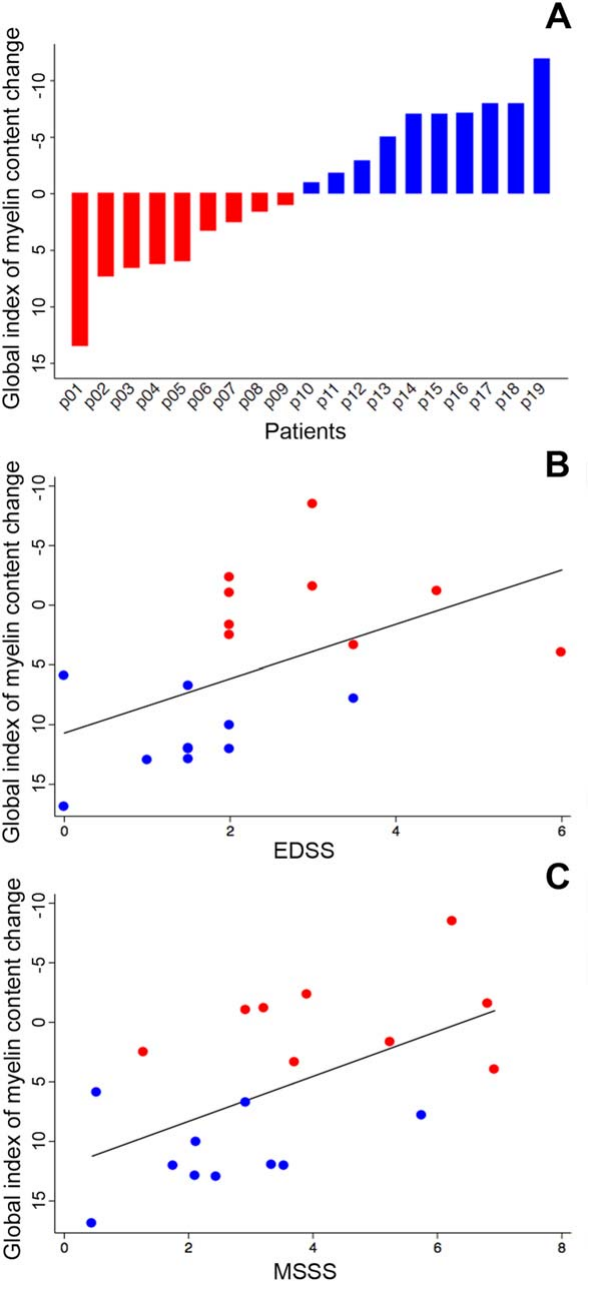
Between‐patient heterogeneity in the global index of myelin content change values. (A) Bar chart diagram displaying the global index of myelin content change value for each patient, which is defined as the difference in demyelinated voxels between the second time point and baseline. This index reflects the individual balance between dynamic demyelination and dynamic remyelination. Patients with positive values on the global index of myelin content change, which indicate a predominant dynamic demyelinating process, are displayed in red. Patients with negative values, characterized by a prevalent dynamic process of remyelination, are indicated in blue. (B and C) Scatter plot diagrams and fitting lines representing the correlations between the global index of myelin content change and clinical scores. Although only a trend toward a significant correlation was found between the global index of myelin content change and EDSS (B), a significant correlation was found between this index and MSSS (C). EDSS = Expanded Disability Status Scale; MSSS = Multiple Sclerosis Severity Scale.

**Figure 4:**
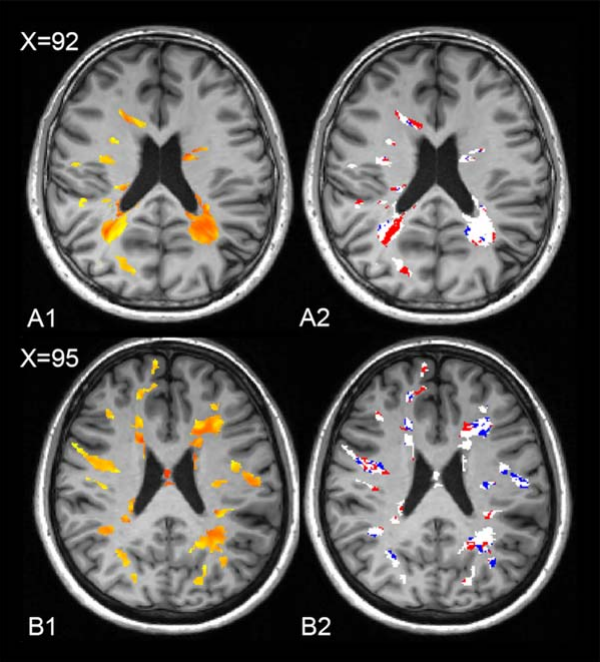
Dynamic myelin loss and regeneration: images from two patients. In A1 and B1, the myelin content of lesional voxels in 2 patients at baseline (patient A: male, 33 years old, disease duration 4 years, EDSS 3; patient B: female, 32 years old, disease duration 3 years, EDSS 0), as measured by Pittsburgh compound B ([^11^C]PiB) binding (voxels in red correspond to the values in the lower range, reflecting more severely demyelinated areas), is represented in red and yellow. In A2 and B2, the longitudinal follow‐up of the same patients is displayed, with the demyelinating voxels over time reported in red and the remyelinating voxels reported in blue. The dynamically demyelinating voxels (in red) were defined as normally myelinated voxels at baseline that were classified as demyelinated at the second time point. Dynamically remyelinating voxels (in blue) were those demyelinated voxels at baseline that reached a myelin level within normal limits at follow‐up. EDSS = Expanded Disability Status Scale.

High between‐patient variability was found, with the global index of myelin content change ranging from –11.91 to +13.46 across the cohort (Fig 3A). The *index of dynamic demyelination* also showed high variability, ranging between 8.4% and 20.5% (representative images in Fig 4; detailed values in Supplementary Table 3). A similar finding was observed for the *index of dynamic remyelination*, with variability ranging between 7.8% and 22.6% (Fig 4; Supplementary Table 3). These results suggest that patients could be classified into two groups: those showing high myelin regeneration potential and those with prevalent dynamic demyelination during the follow‐up period (represented as blue and red bars in Fig 3A).

When the local distribution of dynamically changing voxels was investigated, 85.5% of dynamically demyelinating voxels and 81.7% of dynamically remyelinating voxels were localized at the periphery of T2‐w lesions (defined as the 2‐mm‐thick lesional region inside the border).

### The Extent of Myelin Regeneration Inversely Correlates With Clinical Disability

Because no patients were included during a clinical relapse, and because no significant change was detected in either EDSS or MSSS scores during follow‐up, clinical scores measured at baseline were used for clinical correlations. At baseline, no correlation was found between the percentage of demyelinated voxels over total T2‐w lesion load and clinical scores (EDSS as dependent variable: *p* = 0.111; MSSS as dependent variable: *p* = 0.449; Supplementary Table 4).

The effect of the global index of myelin content change on EDSS only showed a trend toward statistical significance (*p* = 0.045; beta‐coefficient = 0.53; Table 2; significance confirmed with ordinal regression: *p* = 0.04). A significant association independent of gender and T2‐w lesion load was found between MSSS and the global index of myelin content change, with the latter increasing in more disabled patients (*p* = 0.002; beta‐coefficient = 0.69; Fig 3B,C; Table 3; significance confirmed with ordinal regression: *p* = 0.043). A significant association was also detected between MSSS and age, with younger patients showing greater levels of disability (*p* = 0.009; beta‐coefficient = –0.54).

**Table 2:** Effect of the Global Index of Myelin Content Change, Index of Dynamic Remyelination, and Index of Dynamic Demyelination on EDSS After Adjustment for Age, Gender, and total T2‐Weighted Lesion Load

		95% confidence interval					
Dependent Variable: EDSS Score	Coefficient	Lower bound	Upper bound	SE	*t*	*p*	Beta‐coefficient
Global index of myelin content change	0.114	0.0029	0.224	0.051	2.20	0.045*	0.529
Age	–0.045	–0.174	0.083	0.060	–0.75	0.463	–0.176
Gender	–0.390	–1.948	1.167	0.726	–0.54	0.600	–0.128
T2 lesion load	1.15e‐05	–1.14e‐05	3.43e‐05	1.07e‐05	1.08	0.300	0.238
Index of remyelination	–0.215	–0.359	–0.072	0.067	–3.22	**0.006** a	–0.674
Age	–0.054	–0.165	0.058	0.052	–1.03	0.322	–0.207
Gender	–0.245	–1.608	1.119	0.636	–0.38	0.706	–0.081
T2 lesion load	5.27e‐06	–1.47e‐05	2.52e‐05	9.30e‐06	0.57	0.580	0.109
Index of demyelination	0.043	–0.211	0.297	0.119	0.36	0.722	0.100
Age	–0.002	–0.145	0.141	0.067	–0.03	0.978	–0.007
Gender	–0.943	–2.642	0.754	0.791	–1.19	0.253	–0.311
T2 lesion load	9.79e‐06	–1.86e‐05	3.82e‐05	1.32e‐05	0.74	0.472	0.203

*Tests significant at significance level = 0.05.

a Tests remaining significant after correction for multiple comparisons (Bonferroni‐adjusted significance level = 0.017).

CI = confidence interval; EDSS = Expanded Disability Status Scale; SE = standard error.

**Table 3:** Effect of the Global Index of Myelin Content Change, Index of Dynamic Remyelination, and Index of Dynamic Demyelination on MSSS After Adjustment for Age, Gender, and Total T2‐Weighted Lesion Load

		95% confidence interval					
Dependent Variable: MSSS Score	Coefficient	Lower bound	Upper bound	SE	*t*	*p*	Beta‐coefficient
Global index of myelin content change	0.202	0.085	0.318	0.054	3.73	**0.002** ^a^	0.690
Age	–0.189	–0.325	–0.054	0.063	–3.01	**0.009** ^a^	–0.541
Gender	–0.497	–2.134	1.139	0.763	–0.65	0.525	–0.120
T2 lesion load	2.80e‐06	–2.12e‐05	2.68e‐05	1.12e‐05	0.25	0.806	0.043
Index of remyelination	–0.295	–0.468	–0.122	0.081	–3.65	**0.003** a	–0.676
Age	–0.181	–0.316	–0.046	0.063	–2.87	**0.012** a	–0.515
Gender	–0.548	–2.192	1.097	0.767	–0.71	0.487	–0.132
T2 lesion load	–7.14e‐06	–3.12e‐05	1.69e‐05	1.12e‐05	–0.64	0.535	–0.109
Index of demyelination	0.251	–0.042	0.545	0.137	1.84	0.088	0.429
Age	–0.139	–0.303	0.026	0.077	–1.80	0.093	–0.395
Gender	–1.242	–3.201	0.716	0.913	–1.36	0.195	–0.300
T2 lesion load	7.53e‐06	–2.52e‐05	4.03e‐05	1.53e‐05	0.49	0.629	0.115

a Tests remaining significant after correction for multiple comparisons (Bonferroni‐adjusted significance level = 0.017).

CI = confidence interval; MSSS = Multiple Sclerosis Severity Scale; SE = standard error.

**Figure 5:**
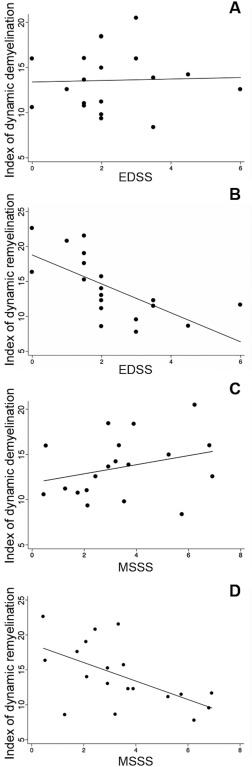
Clinical relevance of remyelination. Scatter plot diagrams and fitting lines representing the correlations between EDSS individual scores and the indices of dynamic demyelination (A) and dynamic remyelination (B) are reported. Although no significant correlation was found between the index of dynamic demyelination and EDSS, a strong inverse correlation was found between the index of dynamic remyelination and EDSS. Patients with lower disability were those presenting higher proportions of remyelinating voxels over total lesion load. Scatter plot diagrams and fitting lines representing the correlations between MSSS individual scores and the indices of dynamic demyelination (C) and dynamic remyelination (D) are also reported. EDSS = Expanded Disability Status Scale; MSSS = Multiple Sclerosis Severity Scale.

Although no significant association was found between the index of dynamic demyelination and EDSS (*p* = 0.72; Fig 5A; Table 2), there was a trend toward a significant correlation with MSSS (*p* = 0.08, beta‐coefficient = 0.43; Fig 5C; Table 3). The index of dynamic remyelination was a significant explanatory factor for EDSS (*p* = 0.006; beta‐coefficient = –0.67, independent of age and gender; Table 2; Fig 5B; significance confirmed with ordinal regression: *p* = 0.001) and MSSS (*p* = 0.003; beta‐coefficient = –0.68; Fig 5D, Table 3; significance confirmed with ordinal regression: *p* = 0.028), together with age (*p* = 0.01; beta‐coefficient = –0.51; Table 3), with patients characterized by higher remyelination potential presenting milder levels of disability. The results remaining significant after correction for multiple comparisons are indicated in Tables 2 and 3 (Bonferroni‐adjusted significance level = 0.017).

When significant regressions were repeated including treatment status as an additional covariate, the effect of the index of dynamic remyelination on EDSS and MSSS (for EDSS: coefficient = –0.233; *p* = 0.014; 95% CI = –0.406 to –0.062; beta‐coefficient = –0.96; for MSSS: coefficient = –0.314; *p* = 0.036; 95% CI = –0.601 to –0.026; beta‐coefficient = –0.796) and the effect of the global index of myelin content change on EDSS remained significant (coefficient = 0.141; *p* = 0.027; 95% CI = 0.020–0.262; beta‐coefficient = 0.952), whereas the effect of the global index of myelin content change on MSSS lost significance (coefficient = 0.165; *p* = 0.108; 95% CI = –0.045 to 0.376; beta‐coefficient = 0.68). No significant effect on either the index of dynamic demyelination or the index of dynamic remyelination was found for gender, disease duration, temporal distance between the two PET scans, volume of Gd+ lesions, and disease‐modifying treatment (Table 5; Supplementary Material), whereas age was a significant contributor to the index of dynamic demyelination only, with older patients showing more‐extensive dynamic demyelination (*p* = 0.014; beta‐coefficient = 0.73; Table 5 in the Supplementary Material).

## Discussion

In this longitudinal study, for the first time to our knowledge, we used high‐resolution [^11^C]PiB‐PET images quantified with a novel non‐invasive approach to visualize and measure lesional myelin loss and regeneration developing over a few months in a cohort of patients with active relapsing‐remitting MS. Cross‐sectional analysis at baseline revealed a decrease in regional [^11^C]PiB uptake that reflected a gradient in myelin concentration from normal‐appearing to lesional tissue. Negative and positive changes in [^11^C]PiB binding were measured in lesions during the follow‐up period, which are suggestive of dynamic myelin loss and regeneration. Finally, strong correlations between the remyelination index derived from [^11^C]PiB PET images and clinical scores were found, supporting the clinical relevance of the remyelination process in patients with MS. Interestingly, no significant effect of the temporal distance between the two PET scans (1–2 months or 3–4 months) on the indices of dynamic demyelination and remyelination was found in this study, which allowed us to analyze all the patients together.

### Cross‐sectional Mapping of Demyelinated Areas on PET Images Reflects the Pathological Distribution of Myelin Loss

The normal range of [^11^C]PiB binding values found in the NAWM of patients with MS may appear contradictory to the changes detected outside visible lesions with several advanced MRI techniques.^5^ However, this finding is in line with histopathological evidence showing that the pathological abnormalities affecting the NAWM mainly consist of axonal damage and loss,^24^ microglial activation,^25^ and disorganized nodes of Ranvier,^26^ but do not include major demyelination. Accordingly, a comprehensive postmortem study combining MRI and histopathology further confirmed that changes in advanced MRI metrics in the NAWM were accounted for by axonal degeneration and microglial activation, but not by demyelination.^27^

Mean binding of [^11^C]PiB was progressively reduced from NAWM to WM lesions, which, in turn, were found to be characterized by a centripetal decrease in [^11^C]PiB binding at the voxel level. These findings mirror postmortem evidence showing that the lesion edge surrounding areas are characterized by a partial decrease in myelin density with an intermediate level between discrete lesions and NAWM,^28^, ^29^, ^30^, ^31^ and that the severity of myelin destruction inside lesions becomes progressively worse from the periphery to the center.^28^

As expected, the lowest mean values of [^11^C]PiB binding were found in the so‐called black holes, characterized by extensive demyelination and axonal loss.^32^, ^33^, ^34^, ^35^ Interestingly, Gd+ lesions showed intermediate [^11^C]PiB binding values between NAWM and T2‐w lesions, which could reflect the initial stage of myelin destruction in lesions that have recently appeared.^32^ Increased permeability of the blood–brain barrier to the tracer in Gd+ lesional tissue could also influence the change in [^11^C]PiB uptake found in active lesions. Exploring this hypothesis would require full modeling of [^11^C]PiB kinetics, including the input function measured with arterial catheterization,^36^ a procedure that we considered too invasive for patients with MS.

### PET With [^11^C]PiB Identifies Individual Profiles in Myelin Regeneration

In our longitudinal analysis, high between‐patient variability was found for all the indices of dynamic myelin content change, particularly the index of dynamic remyelination, reflecting the heterogeneity in remyelination reported in postmortem cases of MS.^37^ This result, if confirmed in larger studies with multiple follow‐up time points, may support the notion of a patient‐specific “remyelination profile,” which determines the extent of myelin regeneration of each individual in response to a demyelinating insult.^37^

Although age had a significant effect on dynamic demyelination and a borderline impact on dynamic remyelination, no significant effect on either index was demonstrated for disease duration. An age‐dependent decrease in remyelination efficiency, possibly resulting from oligodendrocyte progenitor cells migrating and differentiating less efficiently with age in chronic lesions, has been previously highlighted.^38^, ^39^, ^40^ However, the relationship between the efficiency of remyelination, age, and disease duration is still controversial, and our results contribute to an ongoing debate regarding the key question of whether the remyelination potential remains constant throughout life or is modified by aging and disease stage.^37^, ^41^ The majority of voxels dynamically changing their myelin content in either direction during the follow‐up were localized in the peripheral lesional area in accordance to both neuropathological and ultra‐high‐field MRI studies demonstrating that the lesion edge corresponds to the expanding inflammation where new demyelination takes place and to the privileged lesional area where active remyelination occurs.^37^, ^42^

### Dynamic Myelin Regeneration Correlates With Disability Scores

In our patient cohort, the index of dynamic remyelination was strongly associated with clinical scores, suggesting that an efficient remyelination process taking place in an appropriate time window after a demyelinating insult is one of the discriminating factors in determining a better prognosis in MS, at least during the relapsing phase of the disease. Experimental studies have established that remyelination was effective not only during the short‐term recovery of neuronal function, but also, more importantly, in preventing subsequent axonal degeneration, possibly through mechanisms mediated by axon‐myelin interaction.^43^ However, the correlation we found between remyelination and clinical scores may also be influenced by the development of a fast and severe process of axonal degeneration that takes place immediately after acute demyelination in the subgroup of patients with a worse prognosis, which precedes a potential remyelinating process and, in turn, results in a reduced percentage of remyelinating voxels. The spatial and temporal link between myelin repair and axonal damage and loss has to be further explored in future longitudinal studies combining imaging measures specific for myelin and axonal damage. Results from such studies may also clarify whether patients with MS can be defined as “good remyelinators” or “bad remyelinators” throughout their entire disease course, or whether the balance between demyelination and remyelination in each individual changes over time, with one process prevailing over the other at different stages of disease or in different brain areas.

In conclusion, this longitudinal pilot study demonstrates that in vivo imaging of myelin loss and regeneration in MS can be successfully achieved with PET. However, this should be considered an exploratory study, because the number of subjects included was limited. Moreover, although the best available methodology has been applied in this study to minimize the partial volume effect, a possible residual effect of this kind cannot be completely excluded and should be taken into account in the interpretation of our results. As a chronic demyelinating disease occurring early in life, MS is among the most appropriate pathologies in which to investigate the remyelination process over time. Despite the reported limitations, results from this study support that such a regenerative process does occur during the relapsing phase of the disease and might promote neuroprotection and improve clinical prognosis. This imaging approach not only provides novel insight for understanding the pathophysiology of MS, but also provides perspective to enable stratification of patients based on their remyelination potential, thereby allowing clinical trials to be shortened and enabling measurement of the effects of novel drugs targeted at promoting myelin regeneration.

## Author Contributions

B.B., M.V., M.Bo., B.Z., F.T., and B.S. participated in the concept and design of the study. B.B., M.V., D.G.L., M.Ba., E.P., A.C., L.F., C.Lo., M.T., C.P., F.D., C.Lu., M.Bo., F.T., and B.S. performed data acquisition and analysis. B.B., E.P., B.Z., C.Lu., F.T., and B.S. drafted the manuscript and figures.

## Potential Conflicts of Interest

Nothing to report.

## Supporting information

Additional supporting information can be found in the online version of this article.

Supporting Information
