# Supplementary material for: Dynamic Imaging of Individual Remyelination Profiles in Multiple Sclerosis
Source: Ann Neurol. 2016 May 6;79(5):726–38. doi: 10.1002/ana.24620 (PMC5006855; doi:10.1002/ana.24620)
Supplement: Supplementary file 1 — Supporting Information [file ANA-79-726-s001.docx]

**SUPPLEMENTARY APPENDIX TABLE 1. Repetition Time, Echo Time, Inversion Time, Flip Angle, and Pixel Size of the Magnetic Resonance Imaging Sequences Employed in the Study**

| **Sequence** | **Repetition**  **Time**  **(msec)** | **Echo**  **Time**  **(msec)** | **Inversion**  **Time**  **(msec)** | **Flip**  **Angle**  **(degrees)** | **Pixel Size**  (mm) |
| --- | --- | --- | --- | --- | --- |
| 3D-T1w  MPRAGE | 2,300 | 2.98 | 900 | 9 | 1 × 1 × 1 |
| PDw/T2w  TSE | 4,100 | 14/83 | — | 120 | 0.9 × 0.9 × 3 |
| FLAIR | 8,880 | 129 | 2,500 | 120 | 0.9 × 0.9 × 3 |
| T1w SE | 700 | 14 | — | 150 | 1 × 1 × 3 |

3D-T1w MPRAGE = three-dimensional magnetization-prepared rapid gradient‐echo; FLAIR = fast fluid-attenuated inversion recovery; PDw and T2w TSE = turbo spin-echo proton density-weighted and T2-weighed; T1w SE = T1-weighted spin echo.

**SUPPLEMENTARY APPENDIX TABLE 2. Differences in Mean Distribution Volume Ratio Between Normal-Appearing White Matter and Perilesional White Matter, T2-w lesions, Gd+ Lesions, and Black Holes^a^**

| **Independent Variables** | **Coefficient** | **SE** | **95% CI** | ***t*** | ***p*** |
| --- | --- | --- | --- | --- | --- |
| Perilesional  white matter | –0.345 | 0.053 | \| –0.160 \| –0.084 \| \| --- \| --- \| | –6.452 | 1.36e-08 |
| T2-w  lesions | –0.253 | 0.019 | \| –0.291 \| –0.215 \| \| --- \| --- \| | –13.355 | 1.11e-20 |
| Gd+ lesions | –0.119 | 0.020 | \| –0.158 \| –0.080 \| \| --- \| --- \| | –6.083 | 5.72e-08 |
| Black holes | –0.381 | 0.019 | \| –0.420 \| –0.342 \| \| --- \| --- \| | –19.428 | 1.69e-29 |
| Age | –0.0006 | 0.002 | \| –0.005 \| 0.004 \| \| --- \| --- \| | –0.306 | 0.76 |
| Sex | 0.008 | 0.022 | \| –0.039 \| 0.055 \| \| --- \| --- \| | 0.370 | 0.72 |
| Disease duration | 0.002 | 0.002 | \| –0.002 \| 0.006 \| \| --- \| --- \| | 1.05 | 0.31 |
| T2 total lesion load | –3.81e-07 | 3.67e-07 | \| –1.17e-06 4.05e-07 \| , \| \| --- \| --- \| | –1.040 | 0.32 |

^a^Adjusted for age, sex, disease duration, and T2 lesion load.

CI = confidence interval; Gd+ = gadolinium-enhanced; SE = standard error; T2-w = T2 weighted.

**SUPPLEMENTARY APPENDIX TABLE 3.** Index of Dynamic Demyelinationa and of Dynamic Remyelination^b^, Reported for Each Patient

**Patients Index of Dynamic Index of Dynamic**

**Demyelination Remyelination**

| p01 | 20.47 | 7.78 |
| --- | --- | --- |
| p02 | 11.02 | 19.02 |
| p03 | 15.98 | 21.52 |
| p04 | 8.36 | 11.48 |
| p05 | 13.83 | 12.24 |
| p06 | 9.33 | 13.99 |
| p07 | 10.73 | 17.59 |
| p08 | 18.37 | 12.25 |
| p09 | 9.78 | 15.68 |
| p10 | 12.55 | 11.64 |
| p11 | 15.98 | 9.51 |
| p12 | 14.96 | 11.09 |
| p13 | 12.55 | 20.76 |
| p14 | 14.20 | 8.60 |
| p15 | 11.19 | 8.50 |
| p16 | 18.43 | 13.00 |
| p17 | 13.64 | 15.20 |
| p19 | 15.95 | 16.34 |
| p19 | 10.56 | 22.60 |

^a^Defined as the percentage of demyelinating voxels period over total T2 lesion load.

^b^Percentage of remyelinating voxels over total T2 lesion load.

**SUPPLEMENTARY APPENDIX TABLE 4.** Effect of Percentage of Demyelinated Voxels Over Total T2-w Lesion Load Calculated at Baseline on Clinical Scores^a^

| **Dependent Variable:**  **EDSS Score** | **Coefficient** | **95% CI** | **SE** | ***t*** | ***p*** | **Beta-coefficient** |
| --- | --- | --- | --- | --- | --- | --- |
| Percentage of demyelinated voxels at baseline over total T2-w lesion load | –0.069 | -0.156 – 0.018 | 0.040 | –1.70 | 0.111 | –0.436 |
| Age | –0.034 | –0.169–0.101 | 0.063 | –0.54 | 0.597 | –0.133 |
| Sex | –0.942 | –2.46–0.579 | 0.709 | –1.33 | 0.206 | –0.311 |
| T2 lesion load | 1.4e-05 | –1.06e-05–4.01e-05 | 1.8e-05 | 1.25 | 0.233 | 0.306 |

| **Dependent Variable:**  **MSSS Score** | **Coefficient** | **95% CI** | **SE** | ***t*** | ***p*** | **Beta-coefficient** |
| --- | --- | --- | --- | --- | --- | --- |
| Percentage of demyelinated voxels at baseline over total T2-w lesion load | –0.043 | –0.163–0.076 | 0.055 | –0.78 | 0.449 | –0.201 |
| Age | –0.125 | –0.311–0.061 | 0.086 | –1.44 | 0.171 | –0.357 |
| Sex | –1.54 | –3.64–0.544 | 0.975 | –1.59 | 0.135 | –0.375 |
| T2 lesion load | 7.56e-07 | –3.41e-05–3.56e-07 | 1.63e-05 | 0.05 | 0.964 | 0.011 |

^a^After adjustment for age, sex, and total T2-w lesion load.

CI = confidence interval; EDSS = Expanded Disability Status Scale; MSSS = Multiple Sclerosis Severity Scale; SE = standard error; T2-w = T2-weighted.

**SUPPLEMENTARY APPENDIX TABLE 5.** Effect of Age, Sex, Disease Duration, Treatment Status at Study Entry, Temporal Distance in Months Between the Two PET Scans, T2-w, and Gd+ Lesion Load on the Indices of Dynamic Remyelination and Demyelination

**A**

| **Dependent Variable:**  **Index of Dynamic**  **Remyelination** | **Coefficient** | **95% CI** | **SE** | ***t*** | ***p*** | **Beta-coefficient** |
| --- | --- | --- | --- | --- | --- | --- |
| Age | –0.785 | –1.786–0.216 | 0.360 | –2.18 | 0.095 | –0.653 |
| Sex | 1.846 | –8.42–12.114 | 3.698 | 0.50 | 0.644 | 0.205 |
| Disease duration | –0.430 | –1.555–0.694 | 0.405 | –1.06 | 0.348 | –0.357 |
| Treatment | 1.452 | –12.76–15.665 | 5.119 | 0.28 | 0.791 | 0.189 |
| Temporal distance between the two PET scans | 0.409 | –7.192–8.01 | 2.737 | 0.15 | 0.888 | 0.082 |
| T2 lesion load | –0.7e-04 | –3.47e-04–2.05e-04 | 9.9e-04 | –0.71 | 0.515 | –0.517 |
| Gd+ lesion load | 0.003 | –0.003–0.009 | 0.0023 | 1.24 | 0.284 | 0.923 |

**B**

| **Dependent Variable:**  **Index of Dynamic**  **Demyelination** | **Coeff.** | **95% CI** | **SE** | ***t*** | ***p*** | **Beta-coefficient** |
| --- | --- | --- | --- | --- | --- | --- |
| Age | 0.730 | 0.239–1.221 | 0.177 | 4.13 | **0.014** | 0.726 |
| Sex | -4.469 | –9.505–0.567 | 1.814 | –2.46 | 0.069 | –0.595 |
| Disease duration | 0.218 | –0.333–0.770 | 0.199 | 1.10 | 0.334 | 0.216 |
| Treatment | -2.465 | –9.436–4.507 | 2.511 | –0.98 | 0.382 | –0.384 |
| Temporal distance between the two PET scans | 1.315 | –2.413–5.043 | 1.343 | 0.98 | 0.383 | 0.315 |
| T2 lesion load | -3.34e-04 | –1.69e-04–1.02e-04 | 4.87e-05 | –0.69 | 0.530 | –0.291 |
| Gd+ lesion load | -2.8e-04 | –0.003–0.003 | 0.00115 | –0.24 | 0.820 | –0.106 |

CI = confidence interval; Gd+ = gadolinium-enhanced; PET = positron emission tomography; SE = standard error; T2-w = T2 weighted.
